# All-trans retinoic acid suppresses malignant characteristics of CD133-positive thyroid cancer stem cells and induces apoptosis

**DOI:** 10.1371/journal.pone.0182835

**Published:** 2017-08-17

**Authors:** Dan Mei, Bin Lv, Bo Chen, Shan Xiao, Jie Jiang, Yan Xie, Ling Jiang

**Affiliations:** 1 Department of Clinical Medicine, School of Medicine, Shandong University, Jinan, China; 2 Department of General Surgery, Qilu Hospital, Shandong University, Jinan, China; 3 Department of Endocrine and Metabolism, Qilu Hospital, Shandong University, Jinan, China; University of Navarra, SPAIN

## Abstract

Recently, diagnoses of radioiodine-refractory differentiated thyroid cancer (RAI-R DTC) have become more common; prognosis is poor. It has been suggested that cancer stem cells account for radiotherapy resistance. By flow cytometry, different expression percents of CD133 and OCT4 in thyroid cancer cell lines were detected. By real-time quantitative PCR, different mRNA expression of CD133, OCT4, GLUT1, thyroglobulin (TG), thyroperoxidase (TPO) and sodium iodine symporter (NIS) was analyzed; the localization of CD133, OCT4, and NIS expression was examined using immunofluorescence confocal microscopy. Different expression of CD133, OCT4, and NIS in 21 human thyroid cancer and nodule tissues was investigated using immunohistochemistry. CD133-positive cells were isolated by magnetic sorting. Stronger colony formation ability of CD133-positive and weaker ability of CD133-negative cells in vivo were examined by colony formation. The effects of all-trans retinoic acid (ATRA) on CD133-positive cells in vivo were explored with Cell Counting Kit-8, colony formation, apoptosis, cell cycle, and ethynyl deoxyuridine assays. The ARO cell line and RAI-R DTC tissue specimens had more CD133-positive cells. NIS expression was significantly lower in RAI-R DTC tissue compared to radioiodine-sensitive DTC (RAI-DTC) tissue and specimens from patients with thyroid nodule. ATRA inhibited the stem cell characteristics of CD133-positive cells and induced CD133-positive cell differentiation to CD133-negative cells, and promoted CD133-positive cell apoptosis.

## Introduction

Thyroid carcinoma is a very common cancer. Together with follicular thyroid cancer (FTC), papillary thyroid cancer (PTC) is referred to as well-differentiated thyroid cancer (DTC), which constitutes more than 90% of thyroid cancer [1]. Patients with DTC often have a good prognosis, where the 10-year overall survival rates of PTC and FTC are 93% and 85%, respectively [1,2]. However, about 5% of patients with DTC have distant metastasis together with anaplastic thyroid cancer (ATC); where the tumor cells lose the ability to uptake iodine and have poor prognosis, it is referred to as radioiodine-refractory DTC (RAI-R DTC) [3]. RAI-R DTC is resistant to the conventional treatments and has a dire outcome in several months [4,5]. Recent years have seen the proposal of a cancer stem cell (CSC) hypothesis [6], referring to a subset of cells likely responsible for cancer cell self-renewal, proliferation, and dedifferentiation[7,8]. CD133, or prominin-1, is a five–transmembrane domain glycoprotein specifically expressed on the surface of progenitor and hematopoietic stem cells [1]. CD133-positive cells are present in thyroid cancer cell lines and are related with stemness-relevant characteristics [9]. CSCs also express high levels of *OCT4*; the expression of this gene, both in adult stem cells and CSCs, is considered the main regulator of human embryonic stem cell pluripotency and self-renewal capacities [10], which is believed to be the cause of resistance to conventional therapies in cancer [11].

The sodium iodine symporter (NIS) protein is specifically expressed in the thyroid cells, and mediates thyroidal I–transport from the bloodstream to the colloid [12,13,14]. All-trans retinoic acid (ATRA) regulates NIS expression [14]. ATRA induces differentiation in acute promyelocytic leukemia stem cells, and with arsenic trioxide, increases the 5-year survival rate [15,16]. ATRA also has positive effects on cell growth, differentiation, and apoptosis [17,18].

We tested the levels of CD133 expression in BHP10-3, TT2609, and ARO thyroid cancer cell lines and determined the possible relationship between CD133-positive thyroid CSCs and RAI-R DTC. We investigated whether RAI-R DTC has a higher proportion of CD133-positive cells, which would account for the ineffective response to radioiodine therapy. We also examined whether NIS expression is different in RAI-R DTC compared with RAI-DTC, which responds well to radioiodine. Moreover, we explored the latent expression trend between CD133 and NIS, and whether the two were negatively related. Furthermore, we examined the influence of ^131^I on ARO CD133-positive cells, and the effects of ATRA on their growth, differentiation, proliferation, and apoptosis. In this study, we explore possible methods for decreasing the proportion of CD133-positive cells and for increasing NIS expression.

## Materials and methods

### Ethics approval and consent to participate

All tissue samples were collected and analyzed with the prior written informed consent of the patients. Ethics approval was obtained from the Ethic Committee on Science Research of Shangdong University Qilu Hospital (Jinan, China) for the use of clinical materials for research purposes. (IRB No: KYLL-2016-370).

### Cell culture

The thyroid cancer cell lines used in this study were BHP10-3 [19] PTC (kindly provided by Professor Zhao Jiajun; Shengli hospital, Shandong province), TT2609 [20] FTC (kindly provided by Professor LiuZhiyan; Qilu hospital, Shandong province), and ARO [21] ATC (JENNI Biological Technology, Guangzhou). The BHP10-3 and ARO cells were cultured in RPMI 1640 medium containing 10% fetal bovine serum (Gibco, Grand Island, NY, USA), penicillin (100 IU/ml), and streptomycin (100 IU/ml) (Solarbio, Beijing, China). The TT2609 cells were cultured in F12K medium (Gibco) with the same supplementation as the BHP10-3 and ARO cells. The cells were grown at 37°C in 5% CO_2_; the culture medium was changed every 2–3 days. For the experiments, the cells were cultured in RPMI 1640 serum-free medium (SFM) supplemented with basic fibroblast growth factor (bFGF, 20 ng/ml; PeproTech, Rocky Hill, NJ, USA) and epidermal growth factor (EGF, 20 ng/ml; PeproTech).

### Flow cytometry and cell isolation

CD133 and OCT4 expression was evaluated by flow cytometry (FACSCalibur, Becton Dickinson, San Jose, CA, USA). For CD133 analysis, cells were first treated with FcR Blocking Reagent (Miltenyi Biotec, Bergisch Gladbach, Germany), and then incubated in the dark at 4°C for 15 min with phycoerythrin (PE)-conjugated mouse immunoglobulin G 2b (IgG2b) anti-human CD133/2 (clone 293C3, Miltenyi Biotec). For ARO CD133-positive cell magnetic sorting, cells were labeled with 1.0 ml micromagnetic beads conjugated with CD133/1 antibodies, isolated using a magnetic activated cell sorter (MACS) column and isolator (Miltenyi Biotec). Flow cytometry was used as described above to determine CD133-positive cell purity. For OCT4 analysis, cells were fixed and permeabilized with Foxp3 Staining Buffer Kit (eBioscience, San Diego, CA, USA) according to the manufacturer’s instructions. The cells were then incubated with polyclonal rabbit IgG2b anti-human OCT4 (Abcam, Cambridge, UK) at 4°C for 30 min, washed twice with phosphate-buffered saline (PBS), and incubated with fluorescein isothiocyanate (FITC)-conjugated polyclonal goat anti-rabbit IgG (Proteintech, Rosemont, IL, USA). Apoptosis was evaluated using an Annexin V-FITC Apoptosis Kit (BestBio, Shanghai, China). Cells were first washed with PBS twice and then suspended with annexin V buffer. Annexin V was added to the cell suspension, followed by 15-min incubation away from light at 4°C. Then, propidium iodide (PI) was added and the cells were incubated in the same conditions for 5 min, and analyzed by flow cytometry immediately thereafter. Cell cycle was evaluated using a Cell Cycle Kit (BestBio, Shanghai, China) according to the protocol.

### Total RNA isolation and real time-qPCR

Total RNA was extracted and purified from the cultured cells using TRIzol. RNA quantity and quality were assessed by ultraviolet (UV) spectrophotometry. Then, the RNA was reverse-transcribed using a ReverTra Ace qPCR RT Kit according to the manufacturer’s protocol (TOYOBO, Osaka, Japan). *GLUT1*, onfFN, *OCT4*, *TG*, *TPO*, and *NIS* expression was analyzed by PCR (SYBR Green Real-Time PCR Master Mix, TOYOBO). Reactions were carried out at 95°C for 30 s and 40 cycles at 95°C for 5 s, 55°C for 10 s, followed by extension at 72°C for 15 s and termination at 4°C. GAPDH was used as reference. ΔΔCq method was used to analysis the result [22]. The primer sequences are as follows: *NIS* forward 5ʹ-CCCCAGACCAGTACATGCC-3ʹ, reverse 5ʹ-AGCCGAGGTTTGATGAGGTC-3ʹ; *GLUT1* forward 5ʹ-GGCTTCTCCAACTGGACCTC-3ʹ, reverse 5ʹ-CCGGAAGCGATCTCATCGAA-3ʹ; *OCT4* forward 5ʹ-AACCCACACTGCAGCAGATCA-3ʹ, reverse 5ʹ-TCTCGTTGTGCATAGTCGCT-3ʹ; *TG* forward 5ʹ-TGGTTGTTCTGCCTTCCCTC-3ʹ, reverse 5ʹ-TGCTGACATGGGCAACATCA-3ʹ; *TPO* forward 5ʹ-AGGAACTCCCGAGCTGAGAT-3ʹ, reverse 5ʹ-CTGTGAGTATCCCGGCCTTC-3ʹ; onfFN forward 5ʹ-GCTCAAGTGGTCCTGTCGAA-3ʹ, reverse 5ʹ-CCTTCCAACGGCCTACAGAA-3ʹ, GAPDH forward 5ʹ-CAGGAGGCATTGCTGATGAT-3ʹ, reverse 5ʹ- GAAGGCTGGGGCTCATT-3ʹ.

### Immunofluorescence microscopy

Cells were incubated with anti-CD133 antibody (AC133, Abnova, Taipei, Taiwan), followed by incubation with the corresponding fluorescence-conjugated secondary antibody for 1 h at room temperature. Subsequently, the nuclei were stained with diaminophenylindole (DAPI; Beyotime Institute of Biotechnology, Shanghai, China) for 5 min in the dark. Lastly, images were obtained using confocal microscopy. The same procedure was used to examine NIS and OCT4 expression.

### IHC

Paraffin sections were deparaffinized and rehydrated. For antigen retrieval, the slides were boiled in 10 mM citrate buffer (PH 6.0) for 10 min. The slides were incubated with primary anti-CD133 monoclonal IgG1 (AC133, Abnova) overnight at 4°C, followed by incubation with secondary goat anti-mouse antibody; diaminobenzidine (DAB) was used as the chromogen before images were obtained under an optical microscope. The same procedure was used for NIS and OCT4, with their corresponding antibodies. The specimens of RAI-R DTC (n = 7) were selected from 1077 RAI-treatment DTC patients in the time duration of 01.2002–09.2015 according to the criteria of RAI-R DTC [23]. The specimens were collected from Department of Pathology of Qilu hospital. Specimens of 21 patients were evaluated in all the three groups and the participants aged from 20 to 76, with the mean age 47. The quantitation of IHC figures was performed as this paper.[24]

### All-trans retinoic acid treatment

CD133-positive cells were selected and plated in 96-well plates. After 24 h, 5, 10, 20, or 40 uM/l ATRA (Sigma-Aldrich) was added; the same concentrations of dimethyl sulfoxide (DMSO) were added to the respective blank control groups. The cells were observed under a microscope 96 h later. The CCK-8 assay was used to measure the inhibition ratio of cell growth. At the same time point, the EdU assay was conducted. *NIS*, *TG*, *TPO*, *OCT4*, *GLUT1*, and onfFN expression was confirmed by RT-qPCR. Subsequently, the influence of 96 h ATRA treatment on CD133-positive cell apoptosis and division was determined using flow cytometry.

### CCK-8 assay

Cell suspensions were added to 96-well plates and incubated for 24 h. 5, 10, 20, or 40 μM/l ATRA was added to the cells; followed by 96-h incubation. CCK-8 reagent was added and incubated for 2 h. The optical density (OD) was evaluated on the UV absorption spectrum at 450 nm using a microplate reader (Thermo Fisher Scientific, Waltham, MA, USA).

### Colony formation assay

The isolated CD133-positive, CD133-negative, and ATRA-treated CD133-positive cells were incubated in 6-well plates (500 cells in each well) with SFM containing bFGF (20 ng/ml) and EGF (20 ng/ml). During colony growth, the culture medium was replaced every 5 days. Colonies were counted only if they contained >50 cells. Each treatment was carried out in triplicate.

### EdU assay

Cell suspensions (1 × 10^4^ cells) were added to 96-well plates and incubated for 24 h. Subsequently, culture medium was added to the plates, and 5, 10, 20, or 40 μM/l ATRA was added to the cells; the respective control groups were treated with the same amount of DMSO, followed by 2 h incubation. Then, the medium was replaced with fresh medium and 100 μl EdU reagent (RIBOBIO Guangzhou, China) was added and incubated for 2 h. After fixation, 100 ul Apollo reagent was used to each well in room temperature for 30 min. In the end, each well was incubated with DAPI for 30 min and images were obtained under immunofluorescence microscopy.

### Statistical analysis

Three independent experiments were performed, and the data are expressed as the mean ± SD. GraphPad Prism 6 (San Diego, CA, USA) was used for the statistical analyses. Statistical comparisons were based on nonparametric tests and statistical significance was defined at p<0.05. Comparison of multiple means was performed with One-way ANOVA with post-hoc comparison.

## Results

### Identification of CD133-positive cells in thyroid cancer cell lines

**Fig 1:**
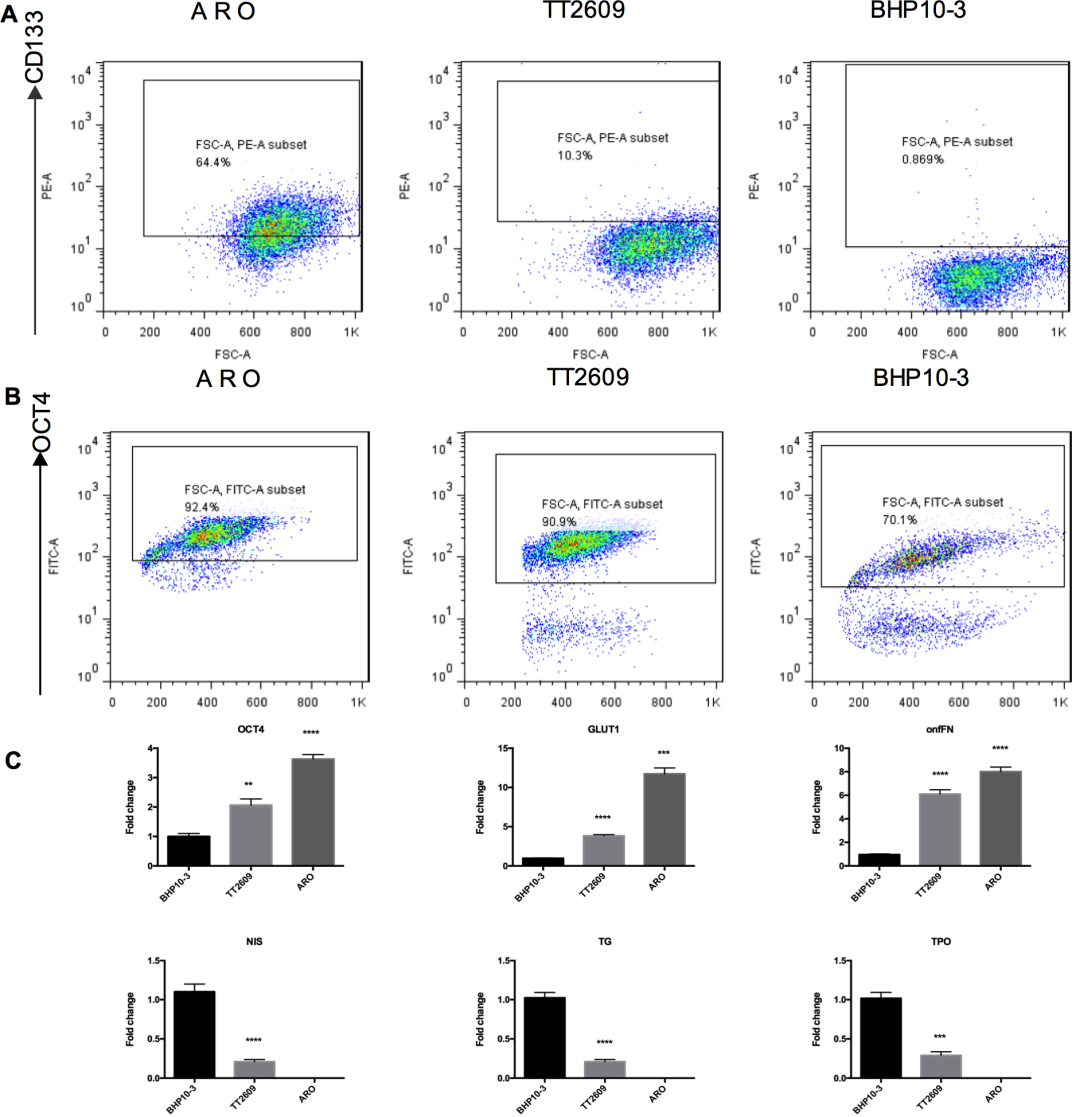
CD133 and OCT4 detection in ARO, TT2609, and BHP10-3 cell lines and RT-qPCR findings. **A.** The proportion of CD133-positive cells in the ARO, TT2609, and BHP10-3 cell lines. **B.** The proportion of OCT4-positive cells in the cell lines. **C.** The relative fold changes of *OCT4*, *GLUT1*, onfFN, *NIS*, *TG* and *TPO* expression (control, BHP10-3 cells).

**Fig 2:**
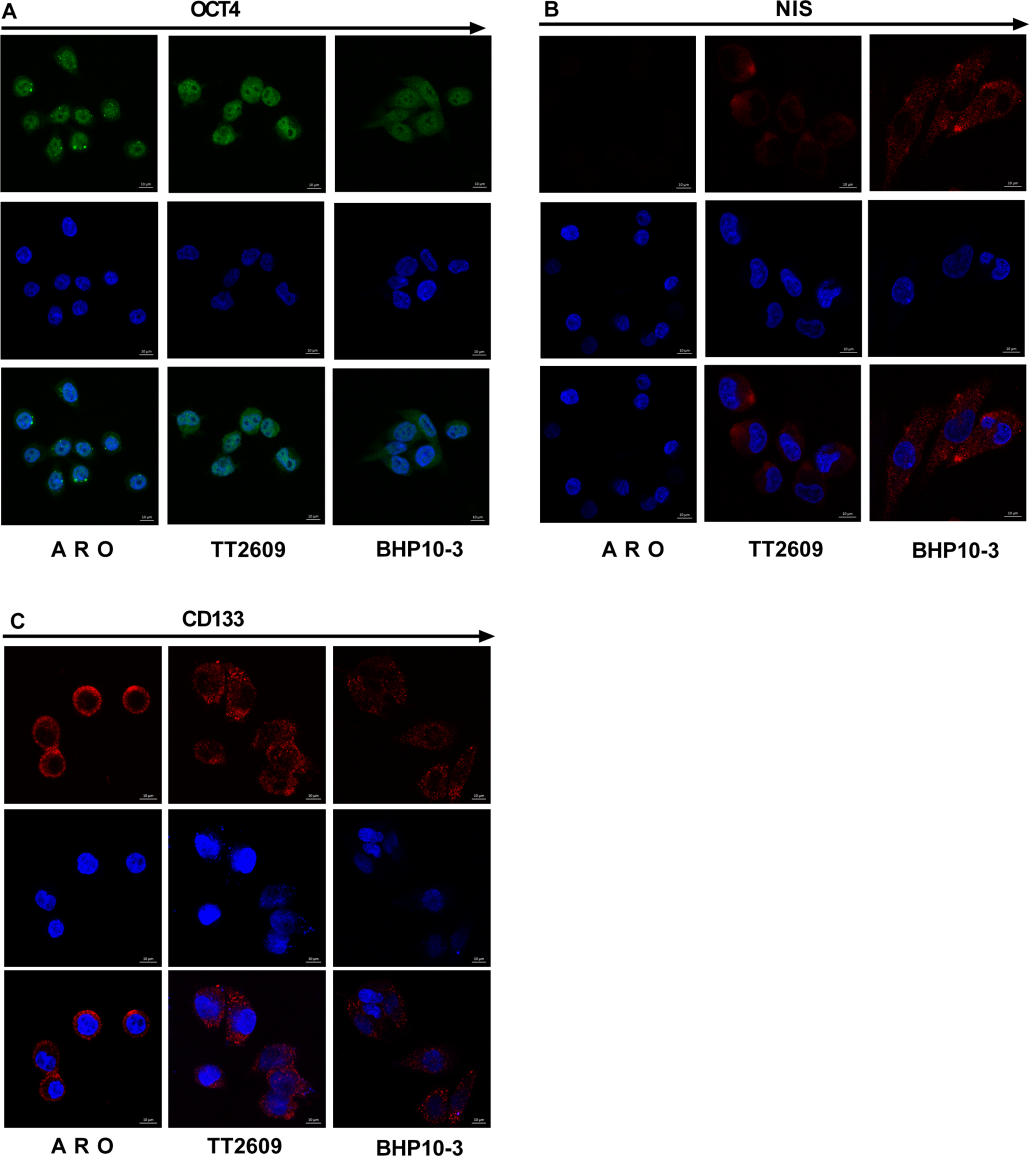
Confocal microscopy detection of CD133, NIS, and OCT4 in ARO, TT2609, and BHP10-3 cell lines. **A.** More and brighter points produced by OCT4 antibody expressed in cell nuclei in ARO and TT2609 cell lines. Less and dimmer points was observed in BHP10-3 cell line. **B.** No NIS expression in ARO cell line; little dim points were observed in cell membrane and cytoplasm in TT2609 cell line and many bright points produced by NIS antibody were observed in BHP10-3 cell line. **C.** More bright points produced by CD133 antibody expressed in cell membrane and cytoplasm were observed in ARO and TT2609 cell lines; less and dimmer points were observed in BHP10-3 cell line.

Flow cytometry determined 64% ± 6%, 10% ± 3%, and <1% CD133-positive cells in the ARO, TT2609, and BHP10-3 cells, respectively. OCT4 was expressed in 92% ± 4%, 90% ± 2%, and 70% ± 7% of ARO, TT2609, and BHP10-3 cells, respectively (Fig 1A and 1B). Confocal microscopy confirmed the flow cytometry results. CD133 in the cells was visible as bright dots in the cell membrane and cytoplasm; OCT4 was visible as bright dots in the nuclei (Fig 2). RT-qPCR confirmed the differentiation status of the cell lines based on the relation between the positive expression tendency of oncofetal fibronectin (onfFN), OCT4, and GLUT1 with the degree of malignancy of the cell lines. The absence of the thyrocyte-specific differentiation markers thyroglobulin (TG), thyroperoxidase (TPO), and NIS in the ARO cell line was also confirmed (Fig 1C and Fig 2).

### Identification of CD133-positive cells in patients with RAI-R DTC

**Fig 3:**
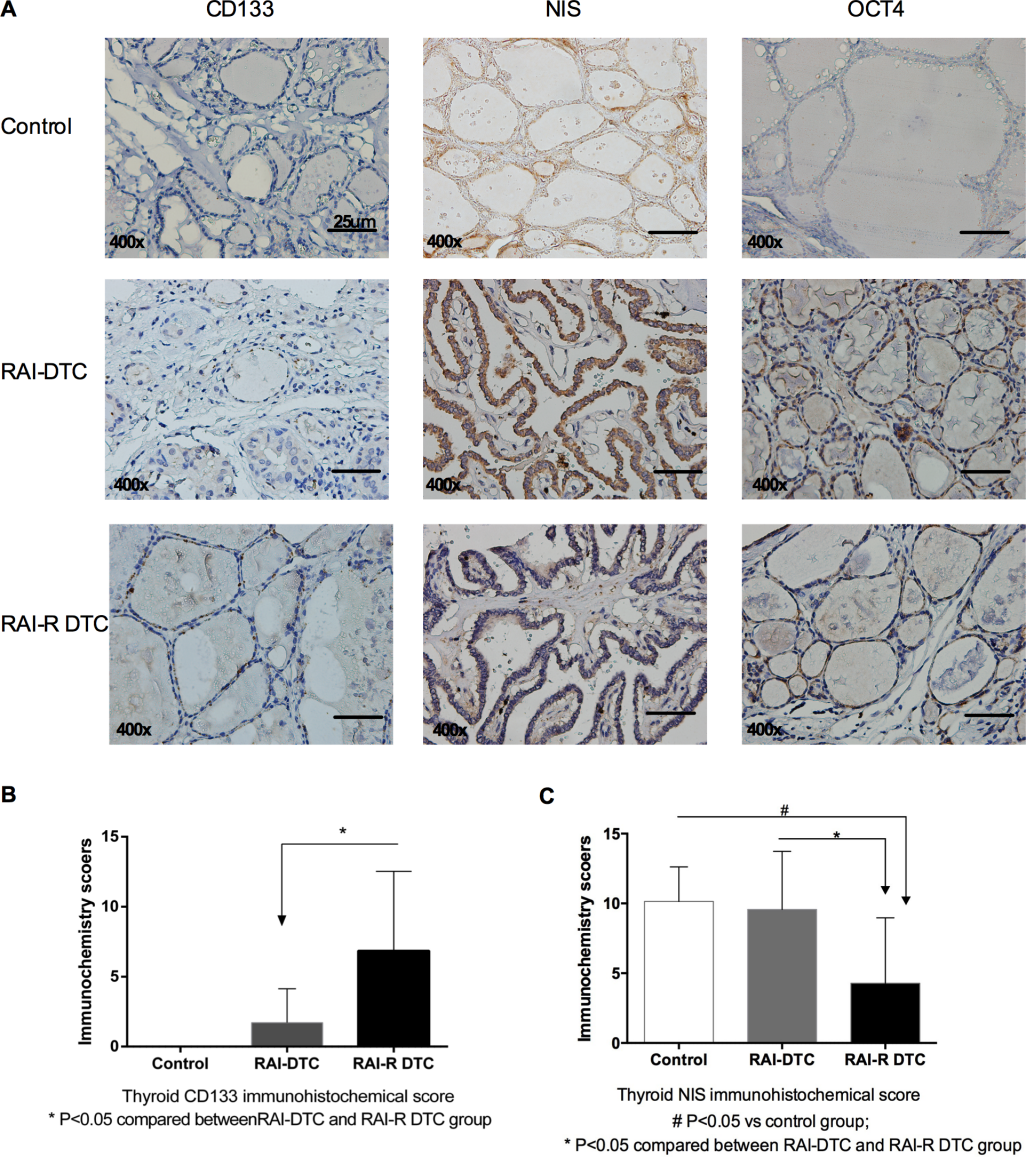
IHC identification of CD133, NIS, and OCT4 in thyroid tumor tissue. **A.** IHC detection of CD133, NIS, and OCT4 expression in the thyroid tumor tissues (400x magnification). **B.** CD133 expression was significantly different between the RAI-DTC and RAI-R DTC groups; *P < 0.05. **C.** NIS expression was significantly different between the three groups; ^#^*P* < 0.05 versus control, *P < 0.05 for RAI-DTC versus RAI-R DTC groups.

Immunohistochemistry (IHC) studies revealed a statistically significant difference in CD133 and NIS expression between the RAI-DTC and RAI-R DTC groups (*P* < 0.05, Fig 3B and 3C). OCT4 expression between the two groups was not significantly different. There was higher CD133 expression and lower NIS expression in the RAI-R DTC group (*n* = 7) as compared to no CD133 expression and high NIS expression in the control group (*n* = 7) and lower CD133 expression and higher NIS expression in the RAI-DTC group (*n* = 7) (Fig 3).

### Magnetic sorting, self-renewal, ^131^I treatment, and colony formation assay of ARO CD133-positive cells

**Fig 4:**
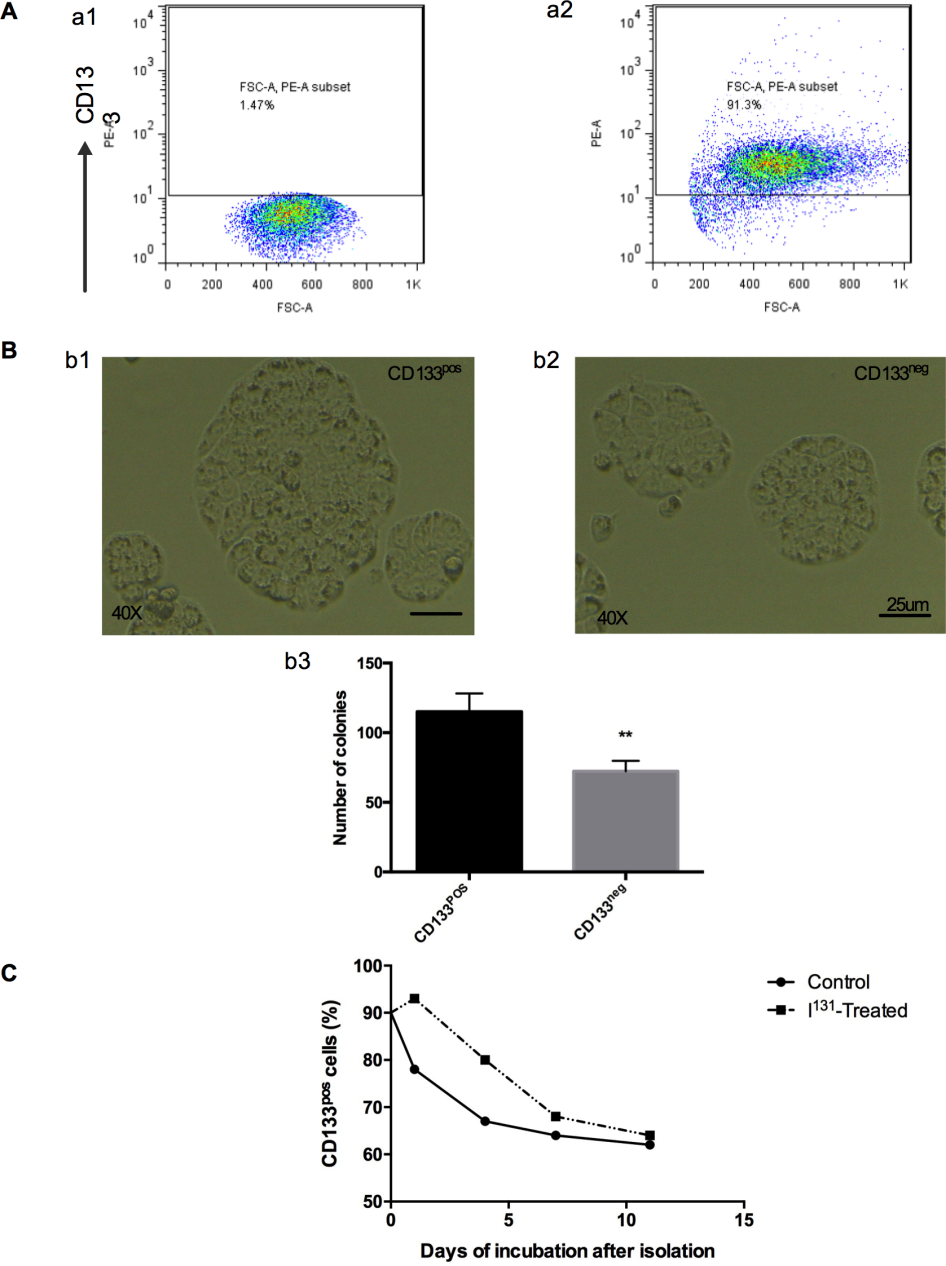
Colony formation assay of ARO CD133-positive and ARO CD133-negative cells, and differentiation of CD133-positive cells with or without ^131^I treatment. **A-a1.** The percent of CD133-positive cells in the isotype control group. **A-a2.** The CD133-positive cell purity after MACS was conducted. **B**-**b3.** The number of colonies after 2-week incubation. **P < 0.01. **B-b1, b2.** The CD133-positive and CD133-negative colonies formed after 5 days. **C.** The proportion of CD133-positive cells after magnetic sorting on day 0, 1, 4, 7, and 11.

Following magnetic sorting, ARO CD133-positive cell purity was as high as 91% (Fig 4A), and the final number of colonies was statistically significant (Fig 4B). CD133-positive cells had a greater proliferative capability than CD133-negative cells: following 5-day incubation after selection, CD133-positive colonies were bigger than the CD133-negative colonies (Fig 4B1 and 4B2). CD133-positive cells are characterized by asymmetrical division, producing one CD133-positive and one CD133-negative daughter cell [9]. ^131^I was added to the culture medium to determine its influence on cell division. Flow cytometry analysis suggested that CD133-positive cells selected after 24-h incubation with ^131^I had higher purity. However, the influence of ^131^I was short-lived, as the purity decreased over subsequent days, with a final purity of approximately 60%, which was similar to that of the original cell line (Fig 4C).

### ATRA treatment of ARO CD133-positive cells

**Fig 5:**
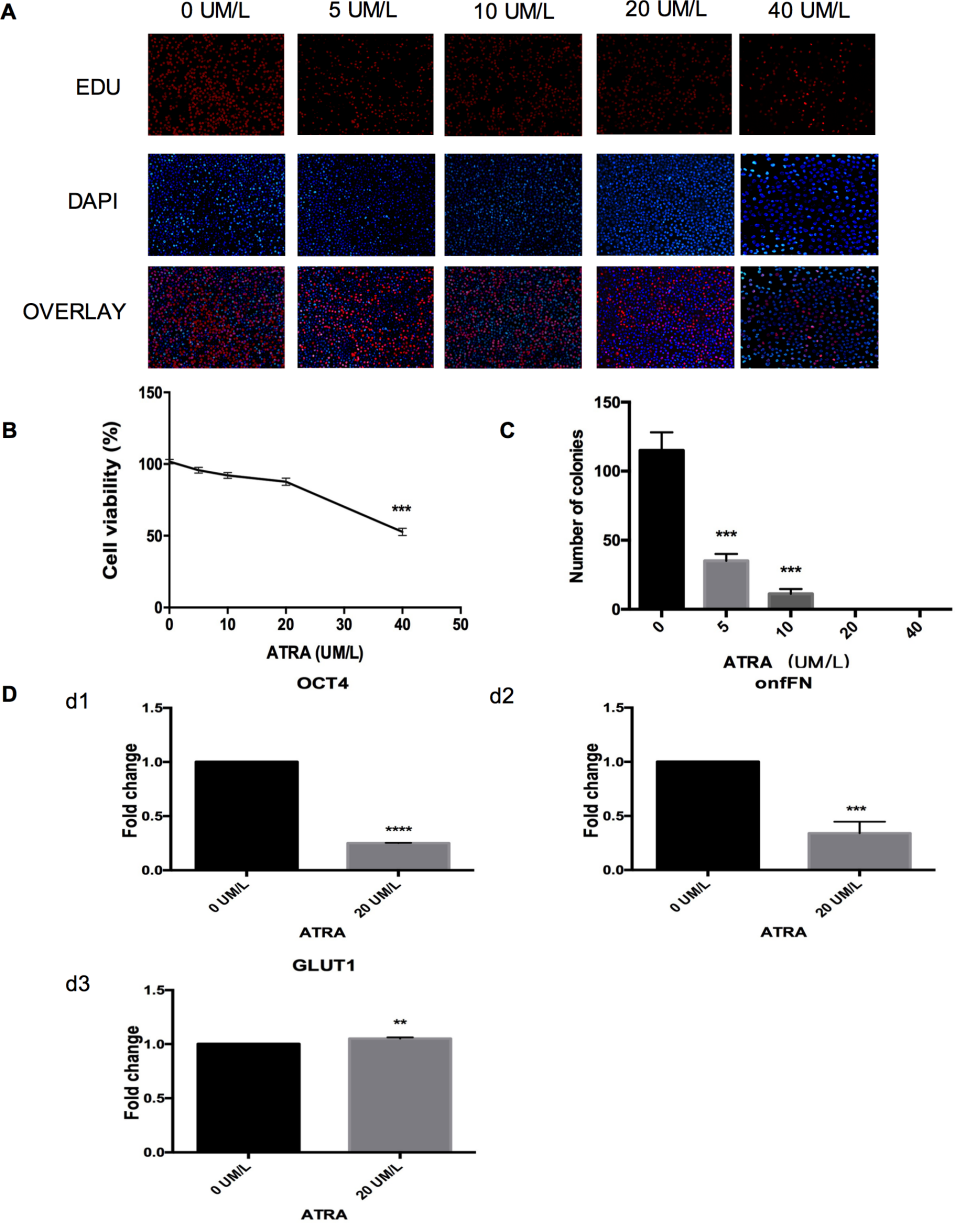
Effects of ATRA treatment. **A.** EdU assay evaluation of MACS isolated CD133-positive cell proliferation after ATRA treatment (40x, scale bar, 25um). **B.** CCK-8 assay evaluation of MACS isolated CD133-positive cell viability after ATRA treatment; ***P < 0.001. **C.** The number of MACS isolated CD133-positive colonies formed after ATRA treatment;***P < 0.001. **D.** RT-qPCR detection of *OCT4*, *GLUT1*, and onfFN expression of MACS isolated CD133-positive cells(control, 0 μM/L ATRA); **P < 0.01, ***P < 0.001, ****P < 0.0001.

**Fig 6:**
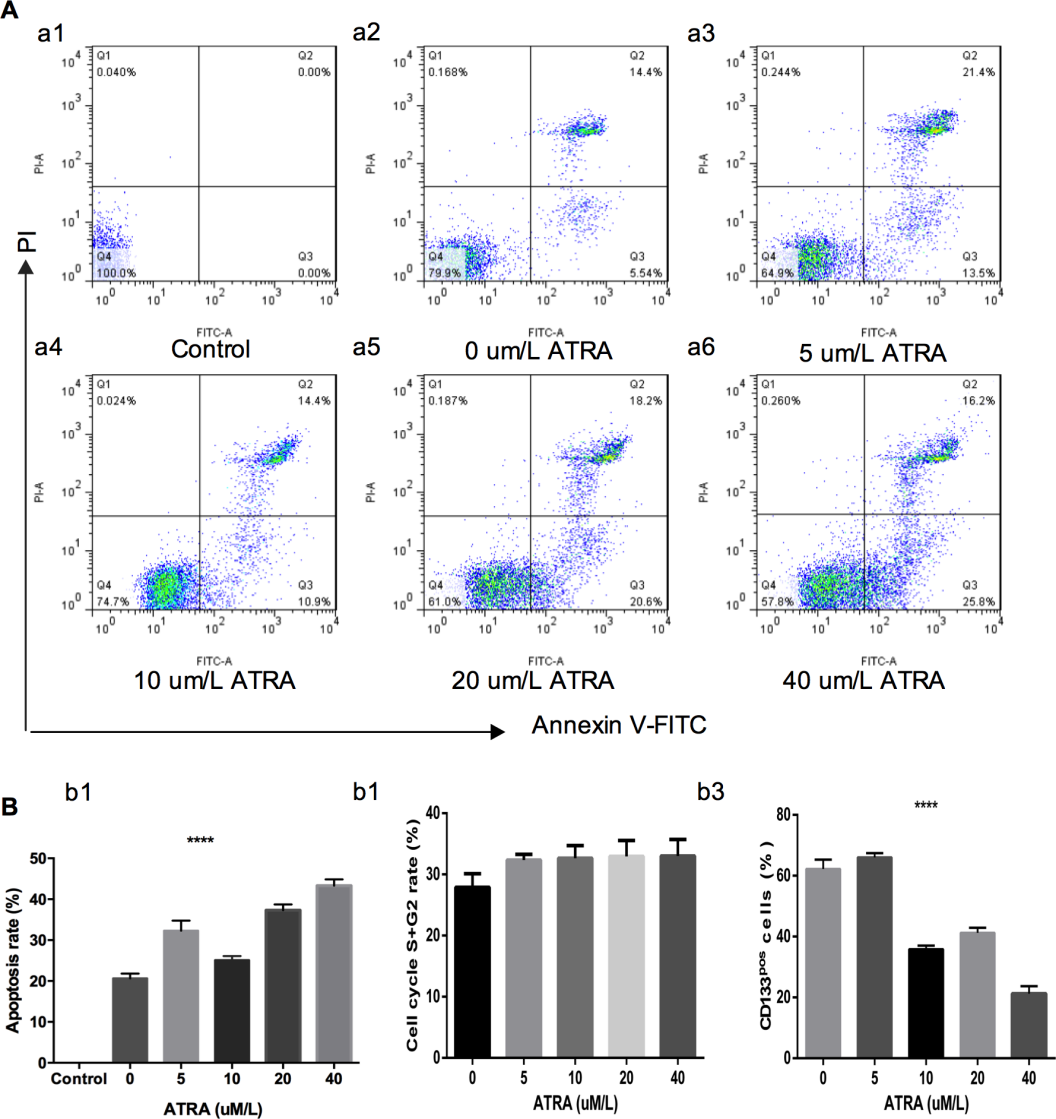
Effects of ATRA treatment. **A.** Flow cytometry analysis of the proportion of apoptosis following ATRA treatment of MACS isolated CD133-positive cells. **B-b1,** The apoptosis rates of CD133-positive cells after ATRA treatment. **b2,** The S+G2 percents after ATRA treatment (P>0.05). **b3** The proportion of MACS isolated CD133-positive cells after 96-h ATRA treatment.

We found that 40 uM/l ATRA significantly inhibited cell proliferation; lower concentrations of ATRA affected no noticeable change (Fig 5A). The Cell Counting Kit-8 (CCK-8) assay confirmed the results. Cell viability was not positively correlated with ATRA concentration. However, the viability of cells treated with 40 uM/l ATRA decreased to approximately half of that of the control (Fig 5B), i.e., the median inhibitory concentration (IC50) was 40 uM/l. The colony formation assay showed that ATRA significantly inhibited the colony formation ability of the CD133-positive cells (Fig 5C). RT-qPCR indicated that the expression of *OCT4*, *GLUT1*, and onfFN, the malignancy genes, was downregulated in CD133-positive cells, while the expression of the thyroid-specific proteins NIS, TG, and TPO was absent (Fig 5D). Except the concentration 5 uM/l, CD133-positive cell apoptosis correlated with ATRA concentration. Similarly, CD133-positive cell division correlated positively with ATRA concentration (Fig 6A, 6B-b1 and 6B-b3). There is a small change in the cell cycle after the ATRA treatment but there is no statistical significance (Fig 6B-b2).

## Discussion

Thyroid cancer is one of the most common cancers and accounts for approximately 95% of the endocrine cancers, and its incidence is increasing [2,25]. ^131^I radiotherapy is the main therapy after surgical resection, and its efficacy is closely related to future recurrence and metastasis. The prognosis of DTC is usually good; however, a small proportion of DTC together with ATC is resistant to radioiodine and has poor prognosis. There is no effective therapy for RAI-R DTC. The CSC hypothesis offers a new insight into cancer progression. Some studies have proved the existence of CSC-like CD133-positive cells in thyroid cancer [26] in the form of a side population according to flow cytometry [27] or by aldehyde dehydrogenase expression [28]. It is believed that CD133-positive cells are CSCs, which are resistant to conventional treatments. Different thyroid cancer cell lines have different proportions of CD133-positive cells [1]. In this study, there was 64% ± 6%, 10% ± 3%, and <1% CD133-positive cells in the ARO, TT2609, and BHP10-3 cell lines, respectively; more CD133-positive cells indicated a higher degree of malignancy. We also examined *OCT4*, *GLUT1*, and onfFN expression by RT-qPCR, and found a positive correlation with differentiation and malignant phenotype (Figs 1 and 2). We also found that CD133-positive cells could survive ^131^I therapy, which might explain why a certain proportion of patients are resistant to radioiodine therapy.

The counterpart expression of several thyroid-specific genes (NIS, TG, TPO) correlates inversely with differentiation and malignant phenotype [29]. The function and regulation of NIS, a thyroid-specific protein, have been well-described, leading to better understanding of thyroid diseases and imaging, and effective radioiodine therapy [30,31]. We also observed the divergent expression of NIS in the three thyroid cancer cell lines, and showed that there is no NIS expression in the ARO cell line, which agrees with the results of previous studies [32]. Our finding that ARO cells do not express NIS might clarify why patients with ATC are insensitive to radioiodine therapy.

In this study, we proved that RAI-R DTC tissues had more CD133-positive cells as compared to RAI-DTC tissue, and had lower NIS expression. Together with the cell line experiment, this result might provide a clearer understanding of the mechanism of RAI-R DTC and provides a new insight into-R DTC (Fig 3). ARO CD133-positive cells undergo asymmetric division and yield one CD133-positive and one CD133-negative progeny [9]. In the present study, CD133-positive cells were enriched after ^131^I treatment, confirming that CD133-positive cells are resistant to radioiodine. This might be the clinical cause of CD133-positive cell enrichment in tumors following ^131^I treatment, as CD133-negative cells would have died and CD133-positive cells would have survived. However, the influence of ^131^I on CD133-positive cell division was short-lived: in the following days, the proportion of CD133-positive cells was same as that in the untreated group. One possible reason for this may be that ^131^I plays a selective role in the external environment without altering the internal cellular mechanisms.

Retinoic acid, bexarotene, and valproic acid have been studied in the context of reinduction of the susceptibility of thyroid cancer to ^131^I therapy [33]. We explored the influence of ATRA on CD133-positive cells, and found that ATRA inhibits their growth, colony formation, proliferation, and division. Furthermore, different concentrations of ATRA had different effects. ATRA promotion of CD133-positive cell apoptosis correlated positively with ATRA concentration. ATRA downregulated the levels of the stemness protein (OCT4), considered a key gene in human induced pluripotent stem cells [34,35,36] together with onfFN and *GLUT1*, although the levels of thyroid-specific proteins (NIS, TPO, TG) were unaltered. We found that 10 uM/l ATRA was the minimum concentration required to alter *OCT4*, *GLUT1*, and onfFN mRNA expression. It has been proven that ATRA induces differentiation [15,37]. We confirm that ATRA induces less CD133-positive cell that correlates with the concentration of ATRA used, but the result of cell cycle assay indicated that there was no meaning change in the cell cycle distribution after ATRA treatment. It was sure that ATRA had the ability to suppress the malignant characters of CD133-positive cells, but the evidence that ATRA could induce redifferentiation of CD133-positive cells was insufficient. These findings might be a possible approach for curing RAI-R DTC.

## Conclusion

The present study confirms that CD133-positive cells play a role in RAI-R DTC. ARO CD133-positive cells are resistant to radioiodine, where this specific population survived ^131^I therapy. ATRA has positive effects on the suppression of the malignant characteristics of CD133-positive cells, although the mechanism is unknown. Next, we intend to research the mechanism that causes CD133-positive cell apoptosis and differentiation following ATRA treatment. Our study provides a new perspective on RAI-R DTC therapy and points to the targeting of CD133-positive cells and the reinduction of NIS expression in the development of a new therapeutic strategy for RAI-R DTC.

## Supporting information

S1 File Cell cycle file. (RAR)

S2 File Quantification of IHC of CD133. (DOCX)

S3 File Quantification of IHC of NIS. (DOCX)

S4 File Data of CCK-8 assay. (PDF)

S5 File PCR file. (XLS)

S6 File PCR file. (XLS)
