# Supplementary material for: All-trans retinoic acid suppresses malignant characteristics of CD133-positive thyroid cancer stem cells and induces apoptosis
Source: PLoS One. 2017 Aug 17;12(8):e0182835. doi: 10.1371/journal.pone.0182835 (PMC5560547; doi:10.1371/journal.pone.0182835)
Supplement: S4 File — (PDF) [file pone.0182835.s004.pdf]

	1	2	3	4	5	6	7	8
**A**		0		5		10		20
**B**		2.649	2.616	2.776	2.698	2.722	2.746	2.809
**C**		2.683	2.583	2.638	2.607	2.648	2.657	2.555
**D**		2.647	2.591	2.655	2.732	2.658	2.615	2.305
**E**		2.566	2.551	2.625	2.315	2.61	2.645	2.087
**F**		2.554	2.562	2.645	2.587	2.666	2.612	2.483
**G**		0.167	0.176	0.172	0.174	0.176	0.169	0.17
**H**						kongbai		

9	10	11	12	
	40			450
2.718	1.884	2.052		450
2.745	1.067	1.423		450
2.235	1.192	1.657		450
2.568	1.018	1.291		450
2.5	1.352	1.102		450
0.171	0.17	0.171		450
				450
