# Supplementary material for: All-trans retinoic acid suppresses malignant characteristics of CD133-positive thyroid cancer stem cells and induces apoptosis
Source: PLoS One. 2017 Aug 17;12(8):e0182835. doi: 10.1371/journal.pone.0182835 (PMC5560547; doi:10.1371/journal.pone.0182835)
Supplement: Supplementary file 2 [file pone.0182835.s002.docx]

Tab. 1 Positive rate and scores of thyroid CD133 immunohistochemical staining

Group	Positive rate of CD133	Scores of CD133
RAI-DTC	42.85%	1.71 ± 0.92
RAI-R DTC	71.43%	6.86 ± 2.14

*P<0.05 compared between RAI-DTC Group and RAI-R DTC Group
