# Supplementary material for: All-trans retinoic acid suppresses malignant characteristics of CD133-positive thyroid cancer stem cells and induces apoptosis
Source: PLoS One. 2017 Aug 17;12(8):e0182835. doi: 10.1371/journal.pone.0182835 (PMC5560547; doi:10.1371/journal.pone.0182835)
Supplement: Supplementary file 3 [file pone.0182835.s003.docx]

Tab. 2 Positive rate and scores of thyroid NIS immunohistochemical staining

Group	Positive rate of NIS	Scores of NIS
Control	100.00%	10.14 ± 0.94
RAI-DTC	100.00%	9.57 ± 1.57
RAI-R DTC	57.14%	4.289 ± 1.77

#P＜0.05 between RAI-R DTC and control group
*P＜0.05 compared between RAI-DTC and RAI-R DTC group
